# The Movable Kidney

**Published:** 1880-11

**Authors:** Herman Mynter


### THE MOVABLE KIDNEY.

FROM THE DANISH BY HERMAN MYNTER, M. D.

Dr. H. P. Oerum has written an interesting monograph on movable kidney, based upon foreign statistics, upon his own experience and upon cases from the clinic of Professor Howitz. In regard to etiology, he mentions abnormal length of the vessels of the kidney, and the presence of a mesentery, but he cannot determine whether it is congenital or developed little by little with the disease. In some cases a great mobility of the colon with strong development of the mesentery was found.

In two post-mortem examinations (by Prosektor Bang) there was found, besides the formation of a mesentery, that the movable kidney was detached from the suprarenal gland, which kept its place; that the fat in the capsule of the kidney had entirely disappeared, and that the small branches from the renal artery to the suprarenal gland were wanting. The author calls attention to the fact, that the movable kidney is seldom found on the left side on account of the distribution of the suprarenal veins, which on the left side empty into the renal vein, while on the right side, they empty directly into the vena cava, and in that way form no connection between the kidney and suprarenal gland. This gland, which is adherent to the diaphragm, scarcely ever takes part in the mobility. The author opposes the opinion of Keppler, who considers movable kidney so serious a lesion, that in most cases nephrotomy is indicated. He believes, that the serious symptoms in Keppler's cases generally may have come from other causes, and that extirpation will be very rarely necessary on account of movable kidney. They consisted in pain in the abdomen, pressure in the epigastrium, chills and vomiting, which occurred in attacks two to three months apart, generally during menstruation. The symptoms lasted two to four days, co-existent with diminished diuresis, and disappeared, when this increased. They were relieved by hot fomentations and subcutaneous injections of morphine. The author explains these symptoms as an acute retention of urine and acute hydronephrosis on account of a sudden dislocation of the kidney, by which the upper part of the ureter is so much bent, that the urine cannot pass. When the hydronephrosis has reached a certain degree, the pressure of the urine will surmount the difficulty. In one case, from the clinic of Professor Howitz, a permanent tumor occurred after the repetition of the acute attacks. In regard to the diagnosis, the author mentions, that an expanded gall-bladder may be mistaken for a movable kidney. The treatment consists in reposition and retention by aid of an abdominal supporter, made of strong linen and provided with elastic straps on the sides, and a pillow under the kidney. If incarceration occurs, reposition is indicated, and if not successful, rest in bed, diet, fomentations and opiates.—*Norkisk Medicinsk Archiv.*

